# The Global Burden of Emerging and Re-Emerging Orbiviruses in Livestock: An Emphasis on Bluetongue Virus and Epizootic Hemorrhagic Disease Virus

**DOI:** 10.3390/v17010020

**Published:** 2024-12-26

**Authors:** Shanta Barua, Eaftekhar Ahmed Rana, M. Asaduzzaman Prodhan, Syeda Hasina Akter, Jully Gogoi-Tiwari, Subir Sarker, Henry Annandale, Debbie Eagles, Sam Abraham, Jasim M. Uddin

**Affiliations:** 1 Faculty of Veterinary Medicine, Chattogram Veterinary and Animal Sciences University, Jakir Hossain Road, Khulsi, Chattogram 4225, Bangladesh; shantabarua007@gmail.com (S.B.); eaftekhar.rana@murdoch.edu.au (E.A.R.); 2 School of Veterinary Medicine, Murdoch University, Perth, WA 6150, Australia; asad.prodhan@murdoch.edu.au (M.A.P.); hasina.akter@murdoch.edu.au (S.H.A.); jully.gogoitiwari@murdoch.edu.au (J.G.-T.); henry.annandale@murdoch.edu.au (H.A.); 3 Biomedical Sciences & Molecular Biology, College of Public Health, Medical and Veterinary Sciences, James Cook University, Townsville, QLD 4814, Australia; subir.sarker@jcu.edu.au; 4 Australian Institute of Tropical Health and Medicine, James Cook University, Townsville, QLD 4811, Australia; 5 Australian Animal Health Laboratory (AHL), Australian Centre for Disease Preparedness (ACDP), East Geelong, VIC 3219, Australia; debbie.eagles@csiro.au; 6 Centre for Biosecurity and One Health, Harry Butler Institute, Murdoch University, Perth, WA 6150, Australia; s.abraham@murdoch.edu.au

**Keywords:** bluetongue virus (BTV), epizootic hemorrhagic disease virus (EHDV), climate change, vectors, vaccination, biosecurity

## Abstract

Bluetongue virus (BTV) and epizootic hemorrhagic disease virus (EHDV) are vector-borne orbiviruses that pose an emerging threat to livestock, including cattle and sheep. This review summarizes the global distribution, genetic diversity, and key factors driving their spread along with the existing knowledge gaps and recommendations to mitigate their impact. Both viruses cause hemorrhagic disease in susceptible ruminants and are commonly reported in tropical and subtropical regions including North America, Asia, Africa, Oceania, and some parts of Europe. The geographical distribution of these viruses, encompassing 27 BTV and 7 EHDV serotypes, has shifted, particularly with the recent invasion of BTV-3, 4, and 8 and EHDV-8 serotypes in Europe. Several factors contribute to the recent spread of these viruses such as the distribution of virulent strains by the movement of temperature-dependent *Culicoides* vectors into new areas due to rapid climate change, the reassortment of viral strains during mixed infections, and unrestricted global trade. These diseases cause significant economic impacts including morbidity, mortality, reduced production, high management costs, and the disruption of international trade. Effective prevention and control strategies are paramount and rely on vaccination, vector control using insecticides, and the destruction of breeding sites, husbandry practices including the isolation and quarantine of infected hosts, restriction of animal movement, prompt diagnosis and identification of circulating strains, and effective surveillance and monitoring plans such as the pre-export and post-import screening of semen used for artificial insemination. However, challenges remain with intercontinental virus spread, live vaccines, and the failure of inactivated vaccines to produce protective immunity against dissimilar strains. Significant knowledge gaps highlight the need for a better scientific understanding and a strategic plan to ensure healthy livestock and global food security.

## 1. Introduction

Bluetongue virus (BTV) and epizootic hemorrhagic disease virus (EHDV) are non-contagious, vector-borne viruses primarily affecting domestic and wild ruminants (Table 1). Both viruses belong to the genus *Orbivirus* within the *Reoviridae* family [1], sharing a close genetic relationship and significant diversity, with multiple serotypes and strains [2]. They contain a segmented double-stranded RNA (dsRNA) genome enclosed in an icosahedral capsid, consisting of ten genomic segments (seg-1 to seg-10) encoding seven structural (VP1 to VP7), and four non-structural proteins (NS1, NS2, NS3/NS3a, and NS4), with VP2 serving as the primary immuno-determinant for viral serotype identification [3,4,5]. Both viruses are listed by the World Organization for Animal Health (WOAH, formerly known as OIE) as notifiable animal diseases due to their global impact [6,7]. To date, a total of 36 BTV serotypes (BTV-1 to BTV-36) have been identified from livestock, among which 27 serotypes are notifiable. Seven EHDV serotypes (EHDV-1, 2, 4, 5, 6, 7, and 8) have been identified from ruminant species [3,4,5,8], of which EHDV-2 known as Ibaraki virus, causes sporadic outbreaks in cattle in Asia [9].

Geographical range and variation in host–vector distribution contribute to the epidemiological differences between these two viruses [2]. Blood-sucking *Culicoides* midges transmit both viruses worldwide [10], with specific species dominating in different regions. BTV is mainly transmitted by *C. imicola* in Africa, *C. insignis* in South America, *C. sonorensis* in North America, *C. brevitarsis*, and *C. wadai* in Australia, and *C. brevitarsis*, *C. wadai*, and *C. fulvus* in Asia and Indonesia [11]. On the other hand, *C. obsoletus*, *C. oxystoma*, *C. imicola*, *C. mohave*, *C. brevitarsis*, and *C. sonorensis* are reported to act as vectors for EHDV [12,13,14,15]. BTV affects a wide range of ruminants, with sheep being the most severely impacted with higher mortality, showing symptoms such as high fever (42 °C), ulcers, and necrosis of the gums, cheeks, and tongue. Severe cases may lead to emaciation, cyanosis of the tongue, coronary band hemorrhage, lameness, abortion, congenital malformation, occasionally pneumonia, and death [16]. Other species like cattle, buffalo, and goats usually show subclinical to mild infection. On the other hand, EHDV predominantly affects domestic and wild ungulates, especially white-tailed deer (WTD) and cattle. EHDV causes similar clinical symptoms as virulent BTV, including high fever, facial edema, difficulty in swallowing or dysphagia, oral ulceration, severe respiratory distress, teat and udder redness, lameness, and decreased milk production in cattle [17,18,19,20,21,22], with occasional abortions, stillbirths, and death [23].

Disease outbreak is a complex and dynamic process involving a susceptible host, virus, vector, human, and environmental factors. The introduction of new serotypes in non-endemic regions is facilitated by inadequate immunization, the failure of the cross-protection of emerging serotypes, improper biosecurity management, mixed farming practices, global warming, vector competence, and illegal global trade [15,24,25]. Orbivirus outbreaks lead to significant economic losses due to increased morbidity, mortality, abortions, stillbirths, fetal abnormalities, reduction in milk, meat, and fleece yield, and restriction of trade, including live animal export [26]. The effective prevention and control of both viruses in livestock depends on a detailed understanding of the complex interaction among host, pathogen, vector, and environment [2], alongside robust surveillance systems and the development of cross-protective vaccines. However, it is challenging to control these diseases due to multiple factors including the importation of live animals and germplasm (such as semen or embryos) through both legal and illegal means, shared host population, the airborne spread of vectors, and the use of inadequately attenuated modified live vaccines [27,28,29]. There are notable knowledge gaps regarding the distribution of vectors and their interaction and survivability with current climate changes, investigations into recombinant vaccines to provide cross-protection against multiple strains of a virus, and effective surveillance and monitoring systems for tracking disease spread across countries. Therefore, this review aims to highlight the global scenario of BTV and EHDV, and identify their contributing factors, along with research gaps and recommendations to enhance prevention and control efforts to reduce the current disease emergence.

## 2. Global Burden of Orbiviruses in Livestock

### 2.1. Bluetongue Virus Infections in Different Geographical Locations

Current phylogenomic analysis illustrates that global BTV strains form two large clades: a Western lineage, with the majority of strains circulating in Africa, the Caribbean, Europe, and the Americas, and an Eastern lineage, circulating in Asia, Indonesia, China, and Australia. BTV was initially confined to South Africa before the 1940s, where 22 of the 27 known serotypes have been identified, and the disease is still endemic there [16]. Until the 1990s, it was mainly found in tropical and subtropical regions including southern Europe [30], but it has since been detected on every continent except Antarctica [30,31,32]. BTV has significantly impacted livestock, with a devastating epidemic between 1956 and 1957 on the Iberian peninsula (Table 2), resulting in 180,000 sheep deaths and a 75% mortality rate [33], and subsequently spread to the Middle East, Asia, North America, and southern Europe [34,35]. BTV also infects water buffalo, with seroprevalence reaching up to 92% in Egypt, Botswana, New Guinea, and India [34]. Various serotypes have been detected in North America and the southeastern United States (Table 3) since 1999 [32,35], mostly confined to their areas of introduction.

In northern Australia including the northern region of Western Australia, BTV-1, 2, 3, 5, 7, 9, 12, 15, 16, 20, 21, and 23 serotypes are detected and serotypes 1, 2, 15, 21 are reported in the eastern states of Queensland (QLD) and New South Wales (NSW) [36,37]. The higher diversity of serotypes in northern Australia is attributed to the migration of *Culicoides* midges from southeast Asia and nearby areas [38]. Genetic analysis of the serotypes detected between 1979 and 1986 [5] (Table 2) revealed that these serotypes were circulating within the Asian–Australasian region, indicating a stable eastern epi-system and possible wind-borne transmission to Australia. Since 2007, four new serotypes (BTV-2, 5, 7, 12) have emerged in Australia, with the last three showing high nucleotide sequence similarity to earlier western topotype isolates [39]. The genome segments of the western genotype of BTV has historically been linked to the European outbreak [31], hinting a potential shift in the eastern epi-system and potential interactions with the northern Australian BTV gene segments. BTV-16, which appeared sporadically from 1984 and re-emerged in 2001, continued to be detected infrequently. Surveillance conducted under the National Arbovirus Monitoring Program (NAMP) revealed widespread BTV-16 transmission in sentinel cattle and sheep with 40% infection rate (per flock), and a 20% mortality rate across north and northwestern NSW in 2023 [40], and by 2024, the strain spread to the south coast and southern tablelands of NSW [41]. Rising temperatures due to climate change in NSW could expand the range of *C. brevitarsis* [42], increasing the risk of BT in livestock and potentially accelerating BTV spread in livestock from the northern to southern zone.

Between 1998 and 2006, BTV primarily affected only part of southern European and the Mediterranean Basin, where its main vector *C. imicola* was prevalent [43], causing over 1.5 million sheep deaths [44,45]. Serotypes 1, 4, 9, and 16 spread into eastern Europe from the Middle East and serotypes 1, 2, 4, and 16 into the western Mediterranean basin from North Africa [43]. Unexpectedly in August 2006, BTV-8 was first documented in the Netherlands [46], marking a significant northward spread (over 900 km). In northern Europe, over 2000 ruminant cases were recorded in the first year of the outbreak, followed by 60,000 and 27,000 cases in consecutive years [47]. This outbreak occurred in regions where the main vector *C. imicola* is rare, suggesting spread by unidentified indigenous European midges like *C. obsoletus*, *C. scoticus*, and *C. dewulfi* [48]. This strain was confirmed to have originated from sub-Saharan Africa [5], with climate change and shifting wind direction affecting vector distribution [49]. The incursion of BTV-8 in France in 2008 was speculated to have derived from the attenuated African vaccine [50]. Simultaneously, genetically similar strains like BTV-6 and BTV-11 were detected in Germany, the Netherlands, and Belgium [51,52] (Table 2), likely introduced to Europe through the illegal use of live attenuated vaccines [32]. These events indicate the expansion of vector range, capacity to distribute the virus in new regions as well as improper and illegal use of live virus vaccines.

In the Netherlands, within-farm transmission of BTV-8 occurred in dairy and sheep flocks during vector season [53], while in France, host movement between distant pastures contributed to the BTV-8 spread in disease-free areas [54]. This serotype caused marked clinical signs and transplacental transmission in cattle and goats [55]. By 2010, an extensive vaccination campaign controlled the outbreak in France [46], although ‘silent circulation’ of the strain continued [56,57], with re-emergence in 2015 [57]. During 2016–2017, the outbreak spread eastward from France, and by 2019, it reached northeast to Switzerland, Germany, and Belgium [58] through movement of infected livestock and *Culicoides* midges [54]. This clearly demonstrates the risk of BTV for global trade, as importing animals from endemic areas without monitoring and improper quarantine can endanger livestock industries. Recently, BTV-8 was again detected in France in August 2023 and identified as a ‘new strain’ causing severe symptoms and mortality [59].

In 2021, BTV were identified in dogs living on a farm in South Africa containing sheep with BT disease [60]. BTV was isolated from cattle and goats in Tunisia in 2022, and in the same year, BTV-4 was reported in mixed sheep and goat herd in Cyprus, which indicates the virus’s wide host diversity. These outbreaks proved that mixed farming, sharing pastures with infected animals, and inadequate husbandry practices could sustain disease circulation, even after vaccination.

**Figure 1:**
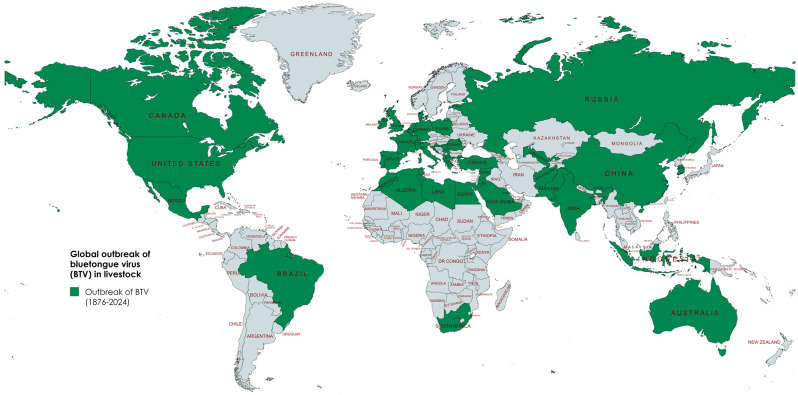
Global outbreak of bluetongue virus (BTV) in livestock from 1876 to 2024.

From 2018 to 2020, the most affected country in the Middle East was Israel, with the detection of nine distinct serotypes [61] (Table 2). BTV-3 reappeared after 60 years in the Mediterranean Basin including Cyprus and Italy [9], likely due to vector transmission or illegal animal movement from North Africa, particularly Tunisia [62,63]. Although BTV-3 showed limited classical signs in sheep in Israel between 2013 and 2017, it emerged as the dominant strain in cattle, sheep, and goat in 2018, attributed to its increased infectivity and adaptability to local vectors. Genome sequencing revealed that Israeli BTV-3 strains (seg-2 and 4) were closely identical to BTV-3 Zarzis/TUN2016 strains from Tunisia [64]. Additionally, Segments 2, 5, 6, 7, and 8 of the Israeli strain were derived from common ancestors, indicating the strain has been circulating in the Mediterranean region since 2013 [64]. In 2021–2022, multiple BTV serotypes were reported in cattle and sheep in Tunisia (Table 2), with heavy rainfall and favorable temperature contributing to vector expansion and outbreaks since 2020 [65]. BTV-3, 4, and 8 were detected in Italy from 2023 to 2024 [66], with BTV-3 likely introduced to Sardinia and Sicily via an infected vector through long-distance wind dispersal from Tunisia [67]. BTV-3 was reported for the first time in continental Europe through an outbreak in the Netherlands in 2023, affecting over 5000 livestock farms, 5996 confirmed cases, and 30–50% mortality rate [68]. The strain spread quickly to Belgium and Germany [66], and by 2024, outbreaks occurred in sheep in France [69], in cattle, sheep, and goats in Luxembourg, in cattle and sheep in Denmark [70], and in cattle and sheep in Germany [71]. Genomic analysis of the VP2 gene of BTV-3 from the Netherlands (BTV-3/NET2023) indicated its origin from the western topotype (Africa, the Mediterranean Basin, and North America), and close relationship with seg-2 BTV-3 isolates from Italy and Tunisia [72]. The quick spread of BTV indicated that indigenous *Culicoides* spp. (although the species has not been identified yet) in the Netherlands effectively transmitted BTV-3/NET2023 [72]. A BTV-4 outbreak in Spain, genetically originated from North Africa [66], continued despite vaccination efforts, with new cases in a previously unaffected area in Spain in 2024 [66]. The sudden emergence of these three strains (BTV-3, 4, and 8) (Table 3) raised concerns about rapid virus transmission in previously unaffected areas from endemic regions, primarily through the short-distance dispersal of *Culicoides* midges and livestock transport within the EU countries. The global outbreak of BTV is shown in Figure 1.

**Table 2:** Historical evidence of BTV infections in diverse species in different countries.

Year	Country/Region	Host	Serotype	References
1876	South Africa	Sheep	Undetected	[73]
1906	South Africa	Sheep	4	[74]
1943, 1969	Cyprus	Sheep	3, 4	[26,75]
1943–1944	Israel	Sheep	Undetected	[76,77]
1950, 1963–1966, 1972–1973	Israel	Cattle, sheep	2, 4, 10, 6, 16	[77]
1948	USA	Sheep	10	[78]
1955, 1962, 1967	USA	Cattle	11, 17, 13	[78]
1956–1957	Iberian Peninsula (Portugal, Spain)	Sheep	10	[26,74]
1959	Pakistan	Sheep	16	[33]
1964, 1967–2000	India	Sheep, goats	1, 2, 3, 4, 6, 9, 16, 17, 18, 23	[79]
1975	Australia	*Culicoides* sp.	20	[80]
1977–1986	Australia	Cattle	1, 3, 9, 15, 16, 20, 21, 23	[81]
1978	Brazil	Domestic ruminants	N/A ^1^	[76]
1979	China	N/A 1	N/A 1	[82]
1979–2011	Australia	Cattle	1	[37]
1981	Indonesia	Sheep	N/A 1	[26]
1982	USA	Cattle	2	[83,84]
2001–2013, 2016, 2017, 2019–2024 (till April 17)	Australia	Cattle, sheep	16	[41,85]
1996	South Africa	Cattle	2, 3, 6, 8	[74]
1998–1999	Greece, Bulgaria, Turkey, Montenegro, Serbia, Macedonia, Kosovo	Cattle, sheep, goat	1, 2, 4, 9, 16	[86]
2000	Tunisia, Algeria, Morocco, Corsica, Sardinia, Sicily	Cattle	2	[87]
2001–2002	Greece, Croatia, Albania, Bosnia, Bulgaria, Republic of Macedonia, Kosovo, Serbia, Yugoslavia	Cattle, sheep, goat	1, 4, 9	[76]
2003–2005	Spain, Portugal, Corsica	Cattle	4	[87]
2004	USA	White-tailed deer	1	[78]
2006–2015	Australia	Cattle	2, 5, 7, 12	[39]
2006–2007	The Netherlands, Belgium, France, Germany, Great Britain, Luxembourg, Denmark, Switzerland, the Czech Republic	Cattle, sheep, goat	8, 6	[88,89,90]
2008	The Netherlands, Germany, Belgium	Cattle, sheep, goat	6, 11	[87,88]
2011	Italy (North Sardinia)	Sheep	8	[88]
2011	Russia	Cattle	14	[91]
2012–2014	Poland	Cattle	14	[91]
2012	Spain, Portugal, Estonia, Russia, Italy	Sheep	1, 2, 4, 8, 9, 14, 16	[48,92]
2013	Russia, Turkey, Jordania	Sheep	14	[48]
2013–2017	Italy	Sheep	1, 4	[88,93]
2014	Balkan countries (Croatia, Greece, Bulgaria, Romania, Slovenia), Portugal, Spain	Cattle, sheep, goat	4	[88]
2015	Australia	Cattle	5, 12	[85,94]
2013, 2016–2020	Israel	Sheep	1, 2, 3, 4, 6, 8, 9, 12, 15	[61]
2014	France	Goats	27	[95]
2014	Balkan countries (Croatia, Greece, Bulgaria, Romania, Slovenia), Portugal, Spain	Cattle, sheep	4	[88]
2015–2019	France	Cattle, sheep, goat	8	[54,96]
2016–2017	France, Sardinia	Cattle	4	[56,88]
2016–2022	Italy	Sheep	3	[62,63,67]
2016–2020	Australia	Cattle, sheep	1	[85]
2017	Greece	Sheep	16	[88]
2017	France, Italy, Spain, Portugal	Sheep	1	[88]
2017–2019	Germany, Belgium, Switzerland	Sheep	8	[88]
2017–2018	France, Italy, Spain, Portugal	Sheep	1	[88]
2020	France, Luxembourg, Belgium, Switzerland, Germany, Spain	Sheep	8	[88]
2020	Greece, North Macedonia, Italy, Romania, Bulgaria, Croatia	Sheep	4	[88]
2018–2020	Israel	Cattle, sheep, goat, wild ruminant	1, 6, 9	[61,97]
2019–2020	Luxemburg, Germany, France, Switzerland, Spain, Belgium	Cattle, sheep	8	[88,96]
2020–2021	Greece, Italy, Romania, Tunisia, Republic of North Macedonia, Bulgaria, Corasia, Spain, France, Italy, Tunisia, Morocco	Cattle, sheep, goat, roe deer, red deer	4	[65,88]
2020–2021	Italy, France	Cattle	1	[88]
2020–2022	Tunisia	Cattle, goat, sheep	1, 2, 3, 4, 26	[98]
2023–2024 (till June)	France	Cattle, sheep	8	[59]
2023–2024 (till August)	The Netherlands, Belgium, England, Germany, UK, France, Denmark	Cattle, sheep, goat	3	[66,69,71,72,98]
2023–2024 (till March)	Italy (Sardinia, Sicily, the mainland)	Cattle	3, 4, 8	[66]
2023–2024 (till March)	Spain	Cattle	4	[66]

N/A ^1^ = data not available.

**Table 3:** Distribution of BTV serotypes and host ranges in different geographical locations.

Regions	Serotype Detected	Host Species	References
African continent (South Africa, Egypt, Algeria, Libya, Morocco, Tunisia and Nigeria)	1, 2, 3, 4, 5, 6, 7, 8, 9, 10, 11, 12, 13, 14, 15, 16, 17, 18, 19, 20, 22, 24, 26	Cattle, buffalo, sheep, goat, alpaca	[74,87,99,100]
European continent (France, the Netherlands, Germany, Italy, Belgium, Spain, Portugal, Switzerland, Ireland, Luxemburg, Denmark)	1, 2, 3, 4, 6, 8, 9, 10, 11, 14, 16, 25, 27	Cattle, sheep, goat, red deer, mouflons, roe deer, fallow deer, Alps	[87,99,101,102]
North American continent (USA, Mexico, Canada)	1, 2, 3, 5, 6, 9, 10, 11, 12, 13, 14, 17, 18, 1 9, 22, 24	Cattle, white-tailed deer, bighorn sheep, mule deer, black-tailed deer, elk, pronghorn, alpaca, mountain goat, bison, blackbuck antelope	[10,16,99,103,104]
South American continent (Brazil, French Guiana, Argentina, Chile, Colombia, Peru, Suriname, Guiana, Venezuela and Ecuador)	1, 2, 3, 4, 6, 7, 8, 9, 10, 12, 13, 14, 17, 18, 19, 20, 21, 22, 24, 26	Cattle, buffalo, sheep, goat, collared peccaries, marsh deer, pampas deer, tapir, guanaco, vicuna	[99,105,106,107,108,109]
Central America and Caribbean region (Guatemala, Honduras, Costa Rica, Panama, Barbados, Trinidad and Tobago, Barbados, Jamaica, Dominican Republic and Martinique, and Guadeloupe)	1, 2, 3, 4, 6, 8, 10, 11, 12, 13, 14, 17, 18, 19, 22, 24	Cattle, sheep, goat,	[99,109]
Australian continent	1, 2, 3, 4, 5, 7, 9, 12, 15, 16, 20, 21, 23, 24	Cattle, sheep, *Culicoides* sp.	[87,99]
South Asia (India, Pakistan, Sri Lanka, Bangladesh, Afghanistan, Bhutan)	1, 2, 3, 4, 5, 6, 7, 8, 9, 10, 11, 12, 13, 14, 15, 16, 17, 18, 19, 20, 21, 23, 24	Cattle, buffalo, sheep, goat, camel, mithun or gayal (*Bos frontalis*)	[34,79,100,110,111,112]
East Asia (China, Japan, and Taiwan, South Korea)	1, 2, 3, 4, 5, 6, 7, 8, 9, 10, 11, 12, 13, 15, 16, 20, 21, 23, 24	Cattle, sheep, goat	[82,100,113]
Southeast Asia (Indonesia and Malaysia)	1, 2, 3, 5, 6, 7, 9, 12, 15, 16, 20, 21, 23	Sheep	[99,113]
Western Asia (Turkey, Cyprus, Syria, Lebanon, Israel, Jordan, Oman, Kuwait, Saudi Arabia)	1, 2, 3, 4, 5, 6, 8, 9, 10, 12, 15, 16, 24, 26	Cattle, sheep, goat	[61,87,114]

### 2.2. Epizootic Hemorrhagic Disease Virus Infections in Different Geographical Locations

EHDV infection has been detected across various continents, including Australia, Africa, North and South America, Asia, and the Middle East, predominantly in tropical and subtropical regions [115]. A new reassortment strain, EHDV-6 (Indiana) was identified in the USA in 2006, combining segments from both Australian EHDV-6 and North American EHDV-2 strains [116], suggesting originated from Australia, and was transmitted through cattle movement. This strain was later found in asymptomatic cattle imported to Trinidad from the USA in 2013. The virus’s ability to reassort and spread via trade highlights its global transmission potential.

The history of EHDV in the ‘Old World’ started in 2003 with the identification of EHDV-6 on Reunion Island (Table 4), though its exact introduction route remains unclear. The virus spreads across several countries, indicating its potential for long distances transmission under favorable conditions. In the USA, EHDV primarily affects farmed WTD; however, a devastating multi-serotype outbreak in 2012 affected cattle across 15 states [24]. EHDV-6 was also isolated from aborted fetuses of cattle, sheep, and goats in Turkey in 2012 and 2017 [117]. Moreover, an outbreak in cattle and sheep was observed in western Turkey, which was postulated to be transmitted by *Culicoides* sp. [117]. In Asia, EHDV-7 caused disease in China [118] and EHDV-1, 2, 7, and 10 affected cattle in Japan (Ibaraki strain, serotype 2) [119], underlining the potential for huge economic losses in the livestock sector and global animal food crisis. Moreover, asymptomatic cases were reported in cattle in several areas: EHDV-6 and 2 in the Caribbean (Martinique, Guadeloupe Islands, and French Guiana), EHDV-1 in French Guiana [120], in Mayotte in 2016 [121], and in western Kenya among local calves from 2007 to 2010 [122] (Table 5). Asymptomatic cases complicate disease diagnosis and control, as infected hosts may act as carriers, facilitating disease transmission in the marketplace and cross-border disease spread.

Recent studies indicated changes in EHDV distribution and epidemiology. The detection of EHDV-2 in WTD and cattle in east-central Canada suggests a possible northward spread of the virus in North America [123,124,125,126]. The host jump and spill-over between species is yet to be fully explained. During the 2021–2022 outbreak, over 200 cases of EHDV-8 were detected in cattle in Tunisia during the vector season [23]. This strain was genetically similar to one previously identified in Australia in 1982 [23]. Notably, BTV outbreak was reported in cattle and sheep in Tunisia in the same year, and both virus outbreaks were attributed to the same vector, *C. imicola*. Current climate change, especially global warming, and the combination of high temperature and rain might have caused vector proliferation to transmit both diseases in the livestock population.

**Figure 2:**
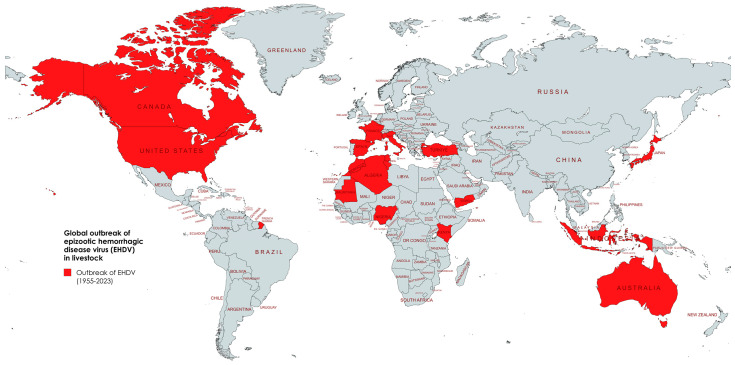
Global outbreak of epizootic hemorrhagic disease virus (EHDV) from 1955 to 2024.

In Europe, EHDV was first reported in deer in Sardinia, Italy in 2022 [127], followed by a second outbreak of EHDV-8 in cattle in Sardinia and Sicily, Italy [127]. The proximity of Sardinia and Sicily to Tunisia (180 km and 140 km, respectively) suggests a potential entry route of the disease into the EU via vectors. The third European territory that suffered from EHDV in cattle was Andalusia, Spain [128], which is located only a few km from Morocco across the Strait of Gibraltar. The serotypes involved in the outbreaks in 2022 in Tunisia, Sardinia, and Andalusia remain unconfirmed. Recent EHDV-8 outbreaks in Spain and Italy followed previous BTV-4 and BTV-3 outbreaks in these countries (Table 5), both believed to have been introduced to Europe by airborne vectors from the Maghreb region (western and central North Africa), with the EHDV-8 genome identical to the strains detected in Tunisia in 2021 [127]. Until November 2023, Spain had reported 125 outbreaks across seven regions affecting cattle and deer [129]. *C. obsoletus* is a significant vector for EHD transmission in Spain [130], and recent outbreaks could be linked to peak *Culicoides* populations, typically seen from mid-summer to late autumn [131]. The disease spread north through the Iberian Peninsula to southern France by 2023 [25], with 3527 outbreaks by November. Interestingly, the first EHDV-positive red deer in France was found dead near a confirmed EHD-infected cattle premises [130]. Exotic hoofed animals like deer and cattle are commonly kept together on hunting reserves [132]. This could increase infection risk due to high animal density, stress, or the presence of non-naïve animals that could amplify the virus, leading to interspecies disease transmission and posing a threat to livestock, trade, and the food supply chain. The global outbreak of EHDV is shown in Figure 2.

**Table 4:** Historical evidence of EHDV infections in diverse species in different countries.

Year	Country/Region	Host	Serotype	Ref
1955	US	White-tailed deer	1	[133,134]
1959–1960, 1997	Japan	Cattle	2 (Ibaraki strain)	[135]
1962, 1987–1988, 1999	Canada	White-tailed deer, cattle, California bighorn sheep	2	[136,137,138]
1967–2970	Nigeria	*Culicoides* sp.	3, 4	[138]
1991	Indonesia	Cattle, Buffalo, Sheep	5	[138]
1992	Australia	Cattle	1, 2, 5, 7, 8	[138]
1993	USA	White-tailed deer	2	[138]
1980–2002	USA	White-tailed deer, mule deer, Beef cattle, pronghorn	1, 2	[138]
2003	Reunion Island, Morocco, Algeria, Tunisia, Turkey, France, the USA, Japan, Trinidad, French Guiana, Maghreb	Cattle	6	[9,19,21,120,139,140,141]
2006–2007	USA, Tunisia, Turkey	Cattle, WTD	6	[116]
2007–2009	USA	White-tailed deer	3	[2]
2012, 2017	Turkey	Cattle, sheep, goat	6	[117]
2013	Trinidad	Cattle	6	[116]
2015, 2016, 2020, 2023	Israel	Cattle	1, 6, 7, 8	[142]
2021–2022	Tunisia	Cattle	8	[23]
2022	Italy (Sardinia)	Deer	Not detected	[127]
2022	Italy (Sardinia, Sicily)	Cattle	8	[127]
2022–2023	Spain	Cattle, deer	Not detected	[128,129]
2023	France	Cattle, deer	3	[25]

**Table 5:** Distribution of EHDV serotypes and host ranges in different geographical locations.

Regions (Country)	Serotype	Host species	Reference
East Asia (China, Japan)	1, 2, 5, 6, 7, 8, 10	Cattle, buffalo, sheep	[15,19,24,118,143,144]
Western Asia (the territory of Bahrain, Oman, Israel, Turkey)	1, 2, 6	Cattle, sheep, goat, wild mountain gazelle	[15,145,146,147,148,149]
Australia	1, 2, 5, 6, 7, 8	Cattle, sheep	[15,150,151]
North America and Canada	1, 2, 6	Cattle, white-tailed deer, mule deer, black-tailed deer, elk, yak, pronghorn, bighorn sheep, bison, blackbuck antelope	[10,15,104,152,153,154,155,156]
South America (Brazil, Colombia, French Guiana, Guiana, Ecuador, Trinidad)	1, 6, 7	Cattle, buffalo, sheep, goat, deer	[15,105,120,157,158]
North Africa (Egypt, Tunisia, Maghreb, West Indies, Libya, Morocco, Algeria) East Africa (Mayotte), West Africa (Nigeria)	1, 6, 8, 4	Cattle, sheep, goat, deer	[22,141,152,159,160,161]
Europe (Italy, Spain, Portugal, France)	6, 8	Cattle, White-tailed deer	[15,22,127]

EHDV and BTV co-infection have been observed in many regions. In 2003, Reunion Island experienced a severe outbreak of both EHDV in cattle [161] and BTV in Merino sheep [162]. A similar co-infection of BTV-2 and EHDV-6 was detected in cattle on Reunion Island in 2009 [139]. In Egypt, BTV-3 and EHDV-1 were isolated from cattle in 2016–17, which were living within the proximity of BTV-positive sheep and goats [148]. In Kenya, seropositive BTV and EHDV cases were found in 51-week-old calves, marking the first detection of EHDV in East African cattle [122]. BTV-9, 13, and 18, and EHDV-1 have been isolated from asymptomatic cattle from farms and slaughter houses in Ecuador [157]. French Guiana identified BTV-1, 2, 6, 10, 12, 13, 17, and 24, and EHDV-1 and 6 in cattle and newly imported sheep and goats in 2011 [120]. The co-circulation of BTV and EHDV reflects a shared epidemiological ecosystem, as both viruses are transmitted by similar *Culicoides* vectors and cause similar symptoms, making the diagnosis of co-exposure challenging. Co-infection raises the risk of genomic reassortment, posing a serious threat to livestock [163]. Favorable climate conditions for vectors, overlapping host populations, and unsafe trade and transportation may lead to co-infection. The circulation of multiple serotypes highlights the need for coordinated monitoring and effective animal movement control.

## 3. Phylogenetic Analysis of BTV and EHDV Sequence

**Figure 3:**
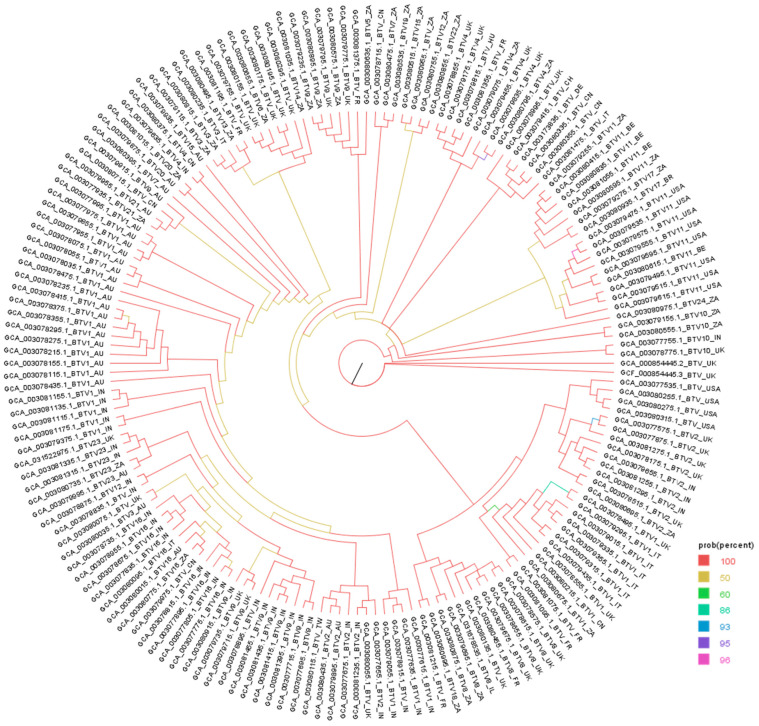
The Bayesian phylogenetic tree of the bluetongue virus (BTV) inferred from 174 BTV genomes that were publicly available in the National Center for Biotechnology Information as of 05 August 2024. The tree branch tips were suffixed with the corresponding country code. AU, Australia; UK, the United Kingdom, USA, the United States; IN, India; ZA, South Africa (Pretoria); CH, Switzerland; TW, Taiwan; DE, Germany; CN, China; HU, Hungary; BR, Brazil; FR, France; BE, Belgium; IT, Italy; IL, Israel. The tree was built using the MrBayes v3.2 [165] program that implemented the ‘invgamma’ model and visualized using the FigTree v1.4.4 [166] software. The colors of the branches represent the posterior probability percentages as indicated in the legend keys. The details of the genomes can be found in Supplementary Table S1.

**Figure 4:**
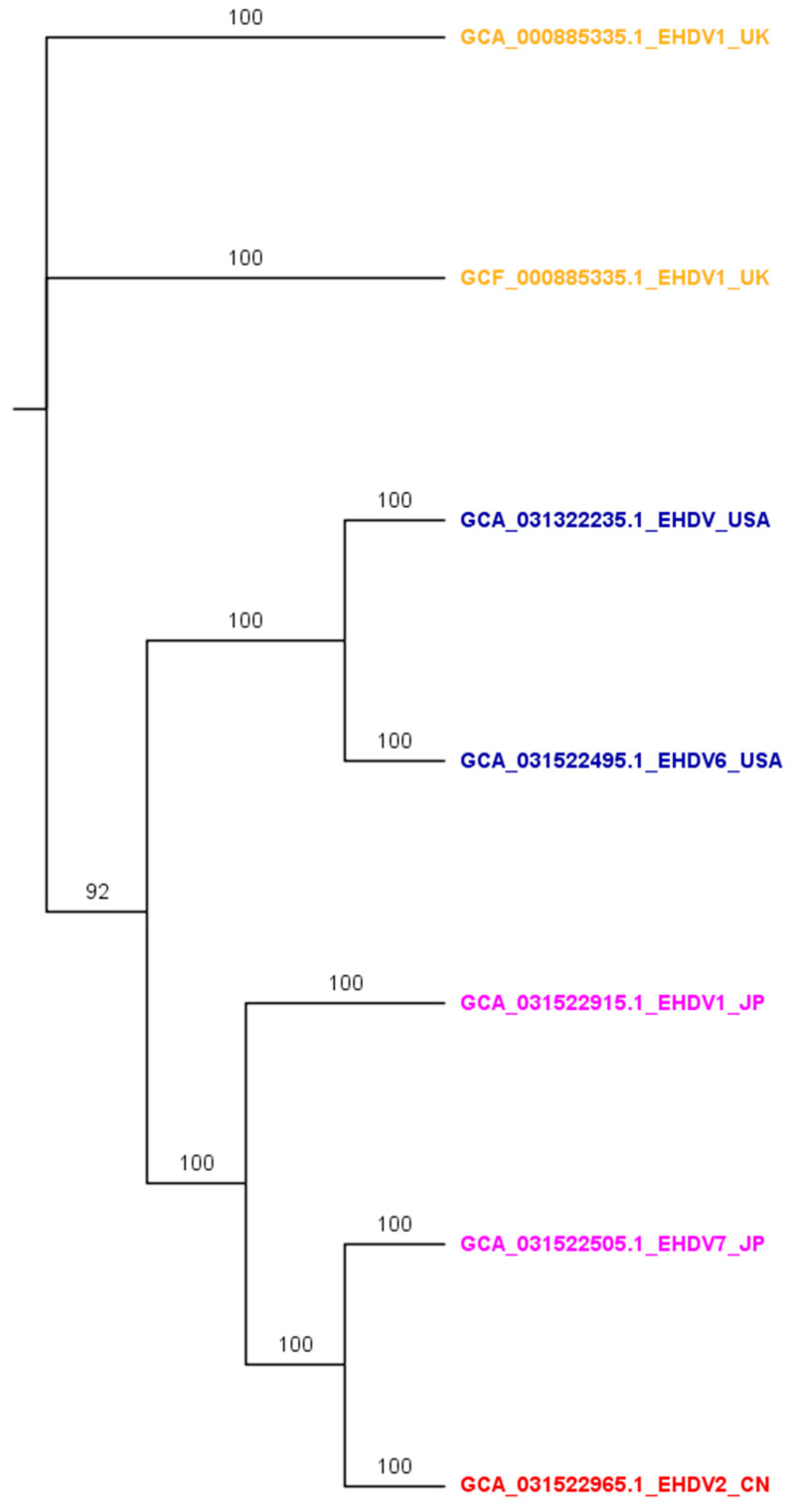
The Bayesian phylogenetic tree of epizootic hemorrhagic disease virus (EHDV) inferred from 7 EHDV genomes that were publicly available in the National Center for Biotechnology Information as of 11 August 2024. The tree branch tips were colored and suffixed with the corresponding country code: UK, the United Kingdom; USA, the United States; CN, China; JP, Japan. The tree was built using the MrBayes v3.2 [165] program that implemented the ‘invgamma’ model and visualized using the FigTree v1.4.4 [166] software. The numbers on the branches represent the posterior probability percentages. The details of the genomes can be found in the Supplementary Table S2.

A total of 174 complete genome sequences of BTV available in the National Center for Biotechnology Information (NCBI) were analyzed to assess the genetic diversity and evolutionary relationships among various isolates or strains obtained from different countries (Figure 3). Most of the sequences clustered closely within the same clades, which generally correspond to their respective countries or geographical regions of origin (Figure 3). In the case of EHDV, only seven complete genome sequences were available in the NCBI database, submitted from four different countries: the United Kingdom, the United States, China, and Japan (Figure 4). Interestingly, the phylogenetic analysis revealed that EHDV sequences from each country formed distinct clades, except the Chinese EDHV sequence nested within the Japanese clade (Figure 4). Our study revealed the regional dominance of certain serotypes: BTV-1 in Australia and Italy, BTV-1 and 16 in India, BTV-11 in the USA and Belgium, and BTV-2 in UK. The roles of viral genetics and proteins at the interface of host susceptibility and the vector in the dynamics of current outbreaks is important. Both BTV and EHDV have a segmented RNA genome, enabling genetic reassortment when multiple strains or serotypes of a virus infect the same host cell or vector that contribute to the emergence of new serotypes or genotypes, enhance the virulence properties, and increase host susceptibility. It is important to note that there is no evidence to support reassortment between two distinct viruses, such as BTV and EHDV. The outer capsid proteins, such as VP2 and VP5, play a critical role in determining host infection. Particularly, VP2 mediates attachment to the host cell receptors, while VP5 facilitates viral entry through host cell membrane penetration. High mutations of the gene and subsequent variations in VP2 among BTV serotypes could increase the affinity of host cell binding and viral entry, thus increasing host susceptibility [151]. Moreover, VP7 interacts with the host’s immune response and provides structural stability that helps the viruses to evade immune response. Additionally, the non-structural proteins like NS3 and NS3A facilitate virus release from infected cells and are subsequently received by *Culicoides* vectors, which impacts viral transmission in susceptible host populations and contributes to new outbreak [164]. It is important to detail sequence and analyze the VP2, VP5, or VP7 genes to understand the mechanism of recent outbreaks of both the viruses. However, it is beyond the scope of this study as we reviewed the burden including genetic relatedness among available genome sequences and vaccines.

## 4. Factors Influencing the High Incidences of BTV and EHDV

Orbiviruses follow seasonal patterns in subtropical and temperate regions, with peak occurrences typically in late summer [167] due to the temperature-dependent activity of the adult *Culicoides* vector. For example, the spread of BTV into northern Europe followed an unusually warm summer in 2006 [43,45]. The distribution of vectors and their coordination with climate determine the disease’s geographic span. The recent introduction of multiple BTV serotypes from the east via Turkey and from the southwest via Africa/Morocco [49,168], which caused an outbreak in previously unaffected areas, was driven by both long-term and short-term climatic changes, expanding the geographic range of *Culicoides* sp. [43,168,169,170]. Alongside ambient temperature, humidity, seasonal rainfall, drought, and wind speed influence the distribution, survival, and breeding of *Culicoides* vectors [171], which thrive in warm, humid, and swampy areas near animal sheds rich in organic matter [26]. For instance, *C. obsoletus* proliferates in wet decaying leaves, water-filled tree cavities, or manure heaps, while *C. dewulfi* breed in the manure heaps of cattle and horses [76]. The combination of severe drought and unusually high temperatures caused significant orbivirus outbreaks during summer of 2012 in USA [126] and BTV infection in big horn sheep in Canada in 2022 [155]. Therefore, a global effort towards the deep sequencing of the isolates and the study of the extensive distribution and survivability of *Culicoides* midges are necessary to understand the transmission dynamics of both viruses.

Recent years have seen significant changes in the global spread of BTV and EHDV due to the reintroduction of infected midges or viremic vertebrate hosts [172]. *Culicoides* can travel up to 5 km or be transported passively over 100 km, particularly over water bodies, aiding in rapid virus spread even in non-endemic regions [26]. BT outbreaks in the Mediterranean, North Africa, Australia, and northern Europe and EHDV dispersal in Israel have been linked to such means [173,174]. The movement of infected livestock, either through legal or illegal ways, increases the risk of virus spread, as evidenced by EHDV-8- and BTV-positive cases in calves imported to Israel from Portugal in 2023 [142].

**Figure 5:**
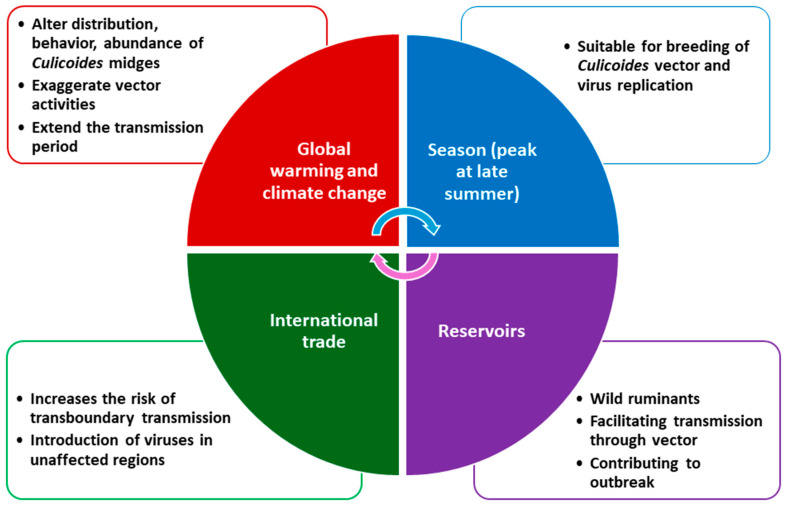
Factors influencing the risk of BTV and EHDV outbreaks.

Global warming is a major driver of BTV and EHDV outbreaks, altering the distribution, behavior, and abundance of the insect vector. Since 1981, global temperatures have risen by over 1 °C, with warming rates exceeding 0.32 °F (0.18 °C) per decade [131,175], prolonging vector survival and extending the transmission period of vector-borne diseases [176]. During BTV-8 and BTV-3 outbreaks in the Netherlands in 2006 and 2023, respectively, temperatures were approximately 2–3 °C higher than the previous 30-year average [68]. Milder winters and overall warming have expanded the range of *Culicoides* midges into previously unsuitable regions in Europe [43]. Previously, EHDV infection was confined to tropical and subtropical regions, and has recently spread to other EU countries. Similarly, summer floods on Australia’s east coast have led to increased recent BTV cases [40]. For example, Australia is one of the leading countries in monitoring BTV through NAMP [41]. The NAMP reports showed that BTV usually circulates in the northern territory, northern western Australia, Queensland, and northern NSW. However, due to global warming and unusual climatic conditions, vectors are spreading south, posing an imminent threat to the sheep industry, located in South Australia, Victoria, and southern western Australia. Nevertheless, detailed studies involving weather parameters, environmental factors, and vector–host dynamics are necessary to better understand the current higher incidences of BTV and EHDV outbreaks. Moreover, wild ruminants, especially WTD and wildebeest, act as important reservoirs for these viruses, often harboring them without visible clinical signs and facilitating transmission through vectors [2]. These reservoirs are key to the persistence and spread of both viruses among domestic animals and in new herd environments, contributing to seasonal outbreaks during periods of high vector activity (Figure 5).

## 5. Economic Impact on Livestock Production and Trade

Determining the economic impact of viral diseases is crucial for planning control strategies. Economic losses can be categorized as direct (production losses) and indirect (expenditure and loss of revenue) [177]. Direct losses include visible impacts like reduced milk production, increased morbidity and mortality, weight loss, decreased fertility, stillbirths, abortion, fetal abnormalities, and reduced meat production efficacy [76]. Indirect losses involve expenditure in disease control and surveillance, diagnostic procedures, vaccination, vector monitoring and control, treatment, and foregone revenue due to trade restrictions [177,178,179]. The overall global agricultural losses due to BTV has been estimated to be approximately 3 billion USD in 1996 [11,74,178]. Livestock mortality rates due to BT and EHD can range from 0 to 100%, impacting regional and national economies depending on outbreak severity [2]. The economic losses reported from various parts of the world are summarized in Table 6.

## 6. Vaccination Against Orbiviruses

Vaccination serves as a pivotal tool in the prevention and management of infection. Since BT vaccines target specific serotypes [182], it is crucial to consider the prevalent serotypes before vaccination in a particular area. Currently, two vaccine types are available against BT, such as live attenuated and inactivated vaccines, while EHDV vaccines have been developed for the countries where the virus has caused significant economic impact.

### 6.1. Vaccines for Bluetongue Virus Disease

Live attenuated vaccines (LAVs) for specific BTV serotypes are used in several countries including the United States, Turkey, South Africa, India, and Israel [78] (Table 7). They are comparatively potent, cost-effective, and offer sufficient protection with a single dose for at least a year. However, LAVs should be administered when vector populations are low to prevent the integration of their genetic material into field BTV strains [183,184]. Since BTV entered Mediterranean Europe, authorities in Spain, France, Italy, and Portugal conducted mandatory vaccination campaigns using modified live attenuated vaccines (MLVs) tailored to the local BTV serotypes from 2000 onwards [183]. Interestingly, MLV vaccines induced strong immunity compared to inactivated vaccines, with trials showing that BTV-2 MLV vaccination prevented viremia in over 90% of cattle for seven months [185]. In field studies, over 80% of vaccinated cattle and sheep maintained detectable levels of BTV antibodies for months [185,186,187], and calves born to vaccinated dams retained colostral antibodies for about 39 days [185]. Following LAV vaccination campaigns in the Balearic Islands in 2000–2003, no outbreaks were reported after December 2003.

Although LAVs are currently used in South Africa and other countries [182] (Table 7), severe side effects have been reported in rams vaccinated with BTV-2 strain produced by South Africa [162]. Vaccination failure with LAVs may occur at temperature above 35 °C and may not protect against other BTV serotypes [176]. Concerns are growing regarding commercial attenuated BTV vaccines due to their adverse effects, such as abortion, reduced milk production, temporary decrease of semen quality in rams [162,188], and fetal malformations in vaccinated pregnant ewes [176,182]. To mitigate these risks, it is recommended to vaccinate ewes 9 to 15 weeks before mating and rams after mating [182], with a minimum of six weeks before the next mating period [188]. There is also concern about potential vaccine reversion to virulence, as observed with BTV-16 MLV [188] [189], which can spread disease across continents through reassortment with a field strain [50]. LAVs fail to eradicate the disease where multiple BTV serotypes co-exist. Therefore, EU countries transitioned from attenuated to inactivated vaccines after 1998 due to concerns over animal welfare and transmission through vectors.

Inactivated vaccines, though more costly, offer long-lasting protection against specific serotypes after one or two doses, helping control epidemics, mitigate direct economic losses, and facilitate safe animal trade [182]. After the 1998 BT outbreaks in southern Europe, monovalent inactivated vaccines were developed for BTV-2 and BTV-4, later followed by bivalent versions [188] (Table 7). Initial weak humoral responses to BTV-2 or BTV-4 vaccines necessities multiple doses, but subsequent doses provide robust and stable protection [190]. After the 2006–2007 BTV-8 outbreak in Europe, a commercial inactivated BTV-8 vaccine was developed (Table 7), offering good efficacy and reducing clinical signs despite requiring multiple doses [191,192]. Moreover, these vaccines prevent both vector and transplacental transmission [191,193,194]. Following the recent BTV-3 outbreak in the Netherlands, Belgium, and Germany, a new inactivated BTV vaccine has been licensed in the Netherlands for emergency use to prevent clinical signs and mortality (Table 7). The field efficacy of inactivated BTV vaccines was demonstrated when over 40,000 vaccinated Spanish cattle tested negative after being in a BTV-affected area [195]. While inactivated vaccines theoretically allow for the differentiation of infected and vaccinated animals (DIVA), this capability has yet to be realized, unlike attenuated live vaccines, which are not suitable for the DIVA test.

It is worth mentioning that inactivated vaccines have inherent significant drawbacks, such as high production costs, the necessity of a large antigenic mass during formulation, slow onset of immunity, and often multiple booster shots [196]. Complex formulations may delay vaccine development and availability [183]. Despite inducing weaker, shorter-lasting immunity than LAVs, inactivated vaccines do not carry the risk of reversion to virulence, gene reassortment with field viruses, or teratogenic effects by crossing the placenta [197].

Currently, novel vaccine types like recombinant vector and subunit vaccines are in development, offering benefits such as eliminating virus transmission risk, rapid immune response, and potential for polyvalent vaccines [182,188]. Recombinant vaccines may provide cross-protection against multiple BTV serotypes, enhancing vaccination strategies. While promising in experimental settings, large-scale field trials are necessary before commercialization [182], which has been limited, possibly due to cost constraints.

### 6.2. Vaccines for Epizootic Hemorrhagic Disease

In Japan, two vaccines for EHDV-2 are available: a monovalent live attenuated vaccine (MLV) and an inactivated bivalent vaccine (Table 8). The EHDV-2 MLV showed high immunogenicity and efficacy [198]. In the USA, where EHDV-1, and EHDV-6 cause recurring outbreaks, autogenous vaccines (Table 8) using inactivated BTV and EHDV antigens are commonly used in sheep and captive cervids [15]. LAVs are not recommended in the EU due to the risk of virus spread through midges or contact transmission [15]. A commercial autogenous killed virus vaccine containing EHDV-1, 2, 6, and 7 strains was administered in a population of captive WTD in the USA, which failed to induce robust antibody response, whereas BTV autogenous vaccines can produce protective antibodies in sheep [199]. The US Department of Agriculture (USDA) has approved bivalent vaccine for EHDV-2 and EHDV-6 for deer in the USA (Table 8). To date, a promising recombinant VP2 subunit vaccine for EHDV-2 [200] has shown strong immunogenicity and protection in WTD [15], inducing high levels of homologous neutralizing antibodies (nAbs) and preventing both disease in host animal and virus transmission to insect vectors. This vaccine is currently undergoing field trials in the USA [15]. Novel vaccines against BTV such as subunit vaccines and viral vector-based vaccines [201] should be explored for developing new EHDV vaccines. The recent EHDV-8 outbreak in the EU is prompting research into next-generation vaccines, emphasizing the need for multi-serotype vaccine approaches due to EHDV’s unpredictable epidemiology. However, the first inactivated EHDV-8 vaccine (vEHDV8-IZSAM) was developed through a study [202], which proved to be safe, immunogenic, and highly effective against the current EHDV-8 strain circulating in Europe in cattle. Collaborative efforts between public veterinary institutions and private pharmaceutical companies are essential for increasing EHDV-8 vaccine production [202].

## 7. Biosecurity and Control Measures

In addition to vaccination, effective control measures are essential for reducing vector-borne orbivirus diseases. While completely eradicating *Culicoides* midges from their natural habitats is challenging, their population can be controlled by stabling susceptible animals overnight, as midges are nocturnal feeders and require blood meals for ovary maturation and egg production [2]. To manage adult midges, measures such as applying approved insecticides, including synthetic pyrethroids (effective for 3–5 weeks) around or inside stables [203], using insecticide-impregnated ear tags, or topical ‘pour on’ treatment, and washing animals with synthetic pyrethroids or organophosphorus (OP) compounds during peak vector seasons are necessary. Aerial pesticide spraying targets adult midges, but treating large areas and free-roaming animals remains challenging. A study in Louisiana revealed higher EHDV/BTV infection rates in cattle rotating through open pastures compared to WTD kept in fenced enclosure [204]. Enhancing stable protection with fine mesh or coarse fabric nets or with synthetic pyrethroid-treated nets further reduces risks [205], while light traps can capture adult midges [26]. For instance, a UV light trap in a sheep pen in 2021 in South Africa successfully captured parous *C. imicola*, the causal vector to transmit BTV between dogs and sheep [60]. In Australia, it was evident that green LED traps significantly attracted the common vectors, especially *C. brevitarsis*, and also newly emerged *Culicoides* sp. affecting livestock [206]. Species like *C. imicola*, *C. obsoletus*, and *C. pulicaris* breed in moist, organic-rich soils that can be drained and dried to eliminate breeding sites of adult midges. Their larval stages can be controlled by draining wastewater lagoons, marshlands, and stagnant water pools, as well as repairing leaks, and shutting off taps to maintain dry conditions [207].

Modifying animal husbandry practices, such as separating affected animals from healthy ones, cleaning stable straws and dung heaps weekly or more frequently [26], and avoiding overcrowding during peak vector activity, can reduce vector exposure. Systemic ivermectin can reduce BT and EHD incidence, while larvicides target midge breeding sites [208]. Insect repellents, such as diethyl toluamide (DEET) ensure protection for up to four hours [176]. Proper insecticide application is crucial to avoid environmental and animal health risks, as high imidacloprid concentration may cause developmental abnormalities in wild deer fawns [209].

Control and prevention programs aim to prevent the virus spread to previously unaffected regions. Veterinary officers, authorities, and livestock farmers must collaborate quickly during outbreaks to prevent further establishments [184,210]. During an outbreak, reliable diagnostic tests like RT-PCR and viral genomic analysis are essential to identify the circulating serotype and infected animals in the herd, which should be promptly managed. However, controlling these infections is challenging by culling due to continuous vector activities and subclinical infection in both domestic and wild ruminants [26]. Affected animals should be retested 7–10 days later to detect any new infections [139]. The movement of susceptible animals should be restricted, and surrounding farms should undergo epidemiological investigations to assess the spread of infection. Strengthening surveillance at borders such as pre-export and post-import monitoring along with screening semen for artificial insemination can reduce the risk of virus introduction. The movement of midges between vaccinated livestock and susceptible wild ruminants could lead to viral evolution and reduce vaccine effectiveness [211]. *C. sonorensis* often feeds on large mammals, and can act as a bridge vector between livestock and wildlife [212]. A survey was conducted for BTV-8 in livestock among five European countries (Denmark, France, the Netherlands, Sweden, and the UK) between 2008 and 2012 [213]. It summarized that detecting emerging diseases is highly region-specific, so active surveillance must incorporate local epidemiological, ecological, and entomological data. Moreover, effective surveillance combining wild ruminants, livestock, and vectors is challenging but crucial to completely eradicate the virus from an endemic region.

## 8. Knowledge Gap and Recommendations

Despite these two important orbiviruses circulating in livestock for decades, there is still limited information about host–pathogen interactions. Understanding disease patterns, global distribution and serotype diversity is of the utmost importance, as is knowledge of the epidemiological zones for studying viral ecology and disease spread. Integrating data on ruminant hosts and *Culicoides* vectors can help predict the expansion of BTV- and EHDV-affected areas. However, viral ecology and evolution present challenges due to the limited availability of genomic and meta-data. Genetic variations in viral genome segments impact their virulence and transmission potential, leading to strain diversity. Further research is needed to understand the evolutionary and molecular processes that enable viruses to infect diverse hosts, and identifying the virulent genetic components and antigenic properties that enhance vector adaptation and facilitate rapid spread is crucial.

Moreover, live animal importation, particularly asymptomatically infected animals from BTV- and EHDV-endemic countries, might act as a potential source of disease outbreaks in naïve populations. Although advanced diagnostic methods make early detection feasible [214], applying these measures during epidemics remains challenging.

The introduction of new virus strains and the rapid evolution of virus serotypes complicates control measures and immunization, as vaccines may lack cross-protection. Therefore, research should focus on developing improved vaccine formulations with broader protection, including recombinant vaccines, which require large-scale field trials to verify safety, efficacy, and cost-effectiveness. Additionally, understanding host immune response to BTV and EHDV infection can aid in the development of vaccines and therapeutic interventions.

BTV and EHDV in wild ruminants are often neglected; they serve as reservoir hosts and can transmit the infection to domestic livestock through vectors under favorable conditions. Research on the movement of *Culicoides* between farms and adjacent wildlife habitats, and interactions between livestock and wildlife can shed light on viral ecology. Integrated surveillance combining sentinel animal studies, serological surveys, and vector monitoring is needed to assess transmission risks of viral serotypes from wild ruminants to livestock and monitor transboundary disease spread. Finally, ongoing monitoring combined with genomic insights into viruses and strong collaboration among livestock producers, animal health researchers, epidemiologists, and decision-makers are highly recommended.

## Figures and Tables

**Table 1:** Summarizing the similarities and dissimilarities between BTV and EHDV.

Similarities	Dissimilarities
Family and Genus: Both BTV and EHDV are belonged to the *Reoviridae* family and are classified under the *Orbivirus* genus. Genome structure: Both viruses have an icosahedral capsid, double-stranded RNA (dsRNA), and segmented genome (ten genomic segments: seg-1 to seg-10), allowing genetic reassortment between strains of the same virus. Proteins: Viral genome encodes seven structural (VP1 to VP7), and four non-structural (NS1, NS2, NS3/NS3a, and NS4) proteins. Outer capsid proteins: Both BTV and EHDV encode the VP2, and VP5 outer capsid proteins, which play a crucial role in virus attachment and entry, serotype determination, antigenic properties, and host immune recognition. Non-contagious nature: None of the viruses spread directly between animals. Vector-borne: Both viruses are transmitted by biting midges, primarily *Culicoides* species. Clinical condition: BTV and EHDV cause similar clinical signs in infected animals, such as fever, hemorrhages, and edema. Geographical distribution and impact: Both viruses are endemic in tropical and subtropical areas. However, the recent outbreak in Europe highlighted the global climate change. Both diseases cause significant economic losses due to decreased productivity, mortality, and trade restrictions.	Genetic diversity: BTV has greater genetic variability with 36 serotypes identified globally, while EHDV has 7 serotypes. Host: BTV: Predominantly causing disease in sheep. The virus can also infect cattle, goats, and deer. EHDV: Primarily affects deer (mainly white-tailed deer). The virus can also infect cattle, sheep, and goats. Vector: Both are transmitted by *Culicoides* midges, but their species vary according to geographical locations. Pathogenic potentiality: Several BTV serotypes cause more severe disease across a range of species compared to EHDV. Morbidity and mortality: BTV: Morbidity is as high as 100%, and mortality 2–30% but can reach to 70%. EHDV: Morbidity and mortality as high as 70% in white-tailed deer. Clinical signs: BTV: Severe symptoms recorded in ruminant livestock, especially in sheep. EHDV: Severe symptoms found in deer, while cattle show mild symptoms. Vaccine: TV: Different serotype specific as well as multivalent commercial vaccines are available for BTV. BEHDV: There is lack of widely used commercial vaccine for EHDV except in Japan.

**Table 6:** Economic impact of BTV and EHDV in different countries.

Country	Year	Types of Animal Herd/Flock	Causes of Economic Loss (Variable Measured)	Estimated Loss (Amount)	References
USA	Yearly (Average)	Cattle, sheep, goat	Trade restriction, diagnosis	144 million USD/year	[177]
France	2007	Dairy cattle	Milk yield reduction	111–249 kg/lactation period	[180]
Germany	2006–2018	Dairy, beef cattle, sheep	Reduced production, mortality, veterinary service, animal export, disease control, vector monitoring	157–203 million Euros (mean 180 million Euros)	[179]
Scotland	2009–2013	Dairy, beef cattle, sheep	Milk yield reduction, weight loss, abortion, infertility, mortality, meat and wool loss, veterinary service, vaccination, carcass disposal, movement restriction, labor cost, surveillance, diagnosis	144 million USD	[178]
TheNetherlands	2006–2007	Cattle, sheep, goat	Milk yield reduction, mortality, early culling, weight loss, postponed gestation, no gestation, abortions, stillbirth, decreased fertility of ram, lower birth weight	In 2006: 32.4 million EUR In 2007: 164–175 million EUR	[177]
Israel	2006	Dairy cattle	Milk yield reduction, mortality, involuntary culling	2,491,000 USD	[181]
Tunisia	2020	Cattle and sheep	Milk yield reduction, mortality, morbidity, veterinary service, weight loss	431.81 to 717.46 million EUR	[65]

**Table 7:** List of bluetongue virus (BTV) vaccines used globally.

Available Vaccine (Trade Name)	Type of Vaccine	Country	Serotype	Target Species	Age (Months)	Dose and Administration	Company Name
Syvazul BTV	Killed	EU	1, 4, 8	Cattle, sheep	Sheep: 3 m Cattle: 2 m	Sheep: 2 mL S/C, booster yearly Cattle: 4 mL I/M, two doses at 3 weeks apart, booster yearly	Syva
BLUEVAC	Killed	Spain	1, 4, 8	Cattle, sheep	Sheep: 2.5 m Cattle: 2 m	Sheep: 2 mL S/C (for serotype 1 and 4, single dose; for serotype 8, double dose) Cattle: 4 mL S/C, two doses at 3–4 weeks apart	CZVACCINES
BLUETONGUE VACCINE	Live	USA	10	Sheep, goat	At weaning time, 3 weeks prior breeding/after lambing	2 mL S/C or I/M	COLORADO SERUM COMPANY
BTV PUR	Killed	France	4, 8	Cattle, sheep	1 m	1 mL S/C, booster after 2/4 weeks	Boehringer Ingelheim
Bovilis BTV8	Killed	France, UK, the Netherlands	8	Cattle, sheep	Sheep: 6 weeks, Cattle: 1 m	N/A ^1^	Intervet
BioBos BTV 8	Killed	Czech Republic	8	Cattle, sheep	1 m	1 mL, 2nd dose-3 weeks later, Booster yearly Cattle: I/M Sheep: S/C	Bioveta, a. s.
RAKSHA-BLU	Killed	India	1, 2, 10, 16, 23	Sheep, goats, buffalo, deer, dromedaries, antelope)	3 m	2 mL S/C, booster yearly	Vea Impex (I) Pvt. Ltd.
BLU-VAX	Killed	South Africa	1, 2, 3, 4, 5, 7, 12, 13, 16, 17, 24	Sheep	N/A 1	1 mL S/C, booster at 3–4 weeks, then yearly	Design Biologix
BLUETONGUE VACCINE FOR SHEEP	Live	South Africa	N/A 1	Sheep	6 m	1 mL S/C, 2nd dose 3–4 weeks apart, booster yearly, 2 months prior to outbreak season	Onderstepoort Biological Products (OBP)
BULTAVO 3	Killed	The Netherlands, UK	3	Cattle, sheep	Sheep: 1 m Cattle: 1 m	Sheep: 1 mL S/C, single dose Cattle: 1 mL I/M, two doses at 2 weeks apart	Boehringer Ingelheim
Syvazul BTV 3	Killed	EU	3	Cattle, sheep	Sheep: 3 m Cattle: 2 m	Sheep: 2 mL S/C, single dose Cattle: 4 mL I/M, two doses at 3 weeks interval	Spanish pharmaceutical company Syva
BLUEVAC-3	Killed	Spain, UK	3	Cattle, sheep	Sheep:2 m Cattle: 2 m	Sheep: 2 mL S/C, two doses at 3 weeks apart Cattle: 4 mL S/C, two doses at 3 weeks apart	Ceva Animal Health in partnership with CZV VACCINES

N/A ^1^ = data not available.

**Table 8:** List of epizootic hemorrhagic disease virus (EHDV) vaccines used globally.

Name of the Vaccine	Type of Vaccine	Country	Serotype
EHDV-2	Monovalent live attenuated vaccine	Japan	EHDV-2
EHDV-2	Inactivated bivalent vaccine	Japan	EHDV-2 and bovine ephemeral fever virus (BEFV)
EHDV vaccine	Autogenous inactivated vaccine	USA	EHDV-1, 2, 6, and BTV-17
EHDV vaccine (CHeRI Lab, University of Florida)	Killed	USA	2, 6

## Data Availability

Not applicable.

## Supplementary Materials

The following supporting information can be downloaded at: https://www.mdpi.com/article/10.3390/v17010020/s1, Table S1: Meta-data of the publicly available bluetongue virus genomes; Table S2: Meta-data of the publicly available epizootic hemorrhagic disease virus (EHDV) genome.
