# Supplementary material for: The Global Burden of Emerging and Re-Emerging Orbiviruses in Livestock: An Emphasis on Bluetongue Virus and Epizootic Hemorrhagic Disease Virus
Source: Viruses. 2024 Dec 26;17(1):20. doi: 10.3390/v17010020 (PMC11768700; doi:10.3390/v17010020)
Supplement: Supplementary file 1 [file viruses-17-00020-s001.zip › viruses-3352064-supplementary.pdf]

Supplementary Materials

Supplementary Table S1. Metadata of the publicly available Bluetongue virus genomes.

Assembly Accession	Assembly Name	Serotypes	Country	Assembly Stats Total Sequence Length	Assembly Release Date
GCA_003078675.1	ASM307867v1	16	Haryana_India	19184	23/09/2013
GCA_003078955.1	ASM307895v1	16	Haryana_India	19183	23/09/2013
GCA_003079055.1	ASM307905v1	1	Haryana_India	19190	23/09/2013
GCA_003079375.1	ASM307937v1	1	Haryana_India	19190	23/09/2013
GCA_003079635.1	ASM307963v1	4	Haryana_India	19176	19/08/2014
GCA_003078735.1	ASM307873v1	16	Haryana_India	19185	24/07/2012
GCA_003079655.1	ASM307965v1	2	Haryana_India	19156	30/11/2012
GCA_003081115.1	ASM308111v1	1	Hisar_India	19188	8/07/2015
GCA_003081135.1	ASM308113v1	1	Hisar_India	19175	8/07/2015
GCA_003081155.1	ASM308115v1	1	Hisar_India	19178	8/07/2015
GCA_003081175.1	ASM308117v1	1	Hisar_India	19190	8/07/2015
GCA_003081235.1	ASM308123v1	2	Hisar_India	19191	8/07/2015
GCA_003081255.1	ASM308125v1	2	Hisar_India	19172	8/07/2015
GCA_003081295.1	ASM308129v1	2	Hisar_India	19202	8/07/2015
GCA_003081315.1	ASM308131v1	23	Hisar_India	19172	8/07/2015
GCA_003081335.1	ASM308133v1	23	Hisar_India	19176	8/07/2015
GCA_003081395.1	ASM308139v1	9	Hisar_India	19175	8/07/2015
GCA_003081415.1	ASM308141v1	9	Hisar_India	19175	8/07/2015
GCA_003081435.1	ASM308143v1	9	Hisar_India	19175	8/07/2015
GCA_003081455.1	ASM308145v1	9	Hisar_India	19175	8/07/2015
GCA_003078575.1	ASM307857v1	8	Pirbright_UK	19201	11/08/2015
GCA_003078615.1	ASM307861v1	8	Pirbright_UK	19201	11/08/2015
GCA_003078655.1	ASM307865v1	8	Pirbright_UK	19202	11/08/2015
GCA_003079755.1	ASM307975v1	N/A[3]	Pirbright_UK	19180	8/06/2010
GCA_003080135.1	ASM308013v1	N/A[3]	Pirbright_UK	19201	8/06/2010
GCA_003080155.1	ASM308015v1	N/A[3]	Pirbright_UK	19180	8/06/2010
GCA_003080175.1	ASM308017v1	N/A[3]	Pirbright_UK	19184	8/06/2010
GCA_003080195.1	ASM308019v1	N/A[3]	Pirbright_UK	19184	8/06/2010
GCA_003080295.1	ASM308029v1	N/A[3]	Pirbright_UK	19175	8/06/2010
GCA_003080075.1	ASM308007v1	N/A[3]	Pirbright_UK	19188	14/05/2012
GCA_003078775.1	ASM307877v1	10	Pirbright_UK	19184	1/05/2012
GCA_003077535.1	ASM307753v1	N/A[3]	USA	19176	19/03/2014
GCA_003080255.1	ASM308025v1	N/A[3]	USA	19196	19/03/2014
GCA_003080275.1	ASM308027v1	N/A[3]	USA	19196	19/03/2014
GCA_003079855.1	ASM307985v1	1	Australia	19195	4/06/2012
GCA_003079875.1	ASM307987v1	20	Australia	19176	4/06/2012
GCA_003079955.1	ASM307995v1	21	Australia	19177	4/06/2012
GCA_003080395.1	ASM308039v1	7	Australia	19155	4/06/2012
GCA_003079995.1	ASM307999v1	23	Australia	19055	4/06/2012
GCA_003079895.1	ASM307989v1	2	Australia	19144	24/05/2012
GCA_003079915.1	ASM307991v1	9	Australia	19104	4/06/2012

GCA_003080435.1	ASM308043v1	2	Australia	19090	4/06/2012
GCA_003079935.1	ASM307993v1	15	Australia	19082	4/06/2012
GCA_003080015.1	ASM308001v1	16	Australia	19053	4/06/2012
GCA_003080035.1	ASM308003v1	3	Australia	18992	4/06/2012
GCA_003077955.1	ASM307795v1	1	Australia	18474	10/10/2014
GCA_003077975.1	ASM307797v1	1	Australia	18474	10/10/2014
GCA_003077995.1	ASM307799v1	1	Australia	18474	10/10/2014
GCA_003078035.1	ASM307803v1	1	Australia	18474	10/10/2014
GCA_003078055.1	ASM307805v1	1	Australia	18474	10/10/2014
GCA_003078075.1	ASM307807v1	1	Australia	18474	10/10/2014
GCA_003078115.1	ASM307811v1	1	Australia	18474	10/10/2014
GCA_003078155.1	ASM307815v1	1	Australia	18474	10/10/2014
GCA_003078215.1	ASM307821v1	1	Australia	18474	10/10/2014
GCA_003078235.1	ASM307823v1	1	Australia	18474	10/10/2014
GCA_003078275.1	ASM307827v1	1	Australia	18474	10/10/2014
GCA_003078295.1	ASM307829v1	1	Australia	18474	10/10/2014
GCA_003078355.1	ASM307835v1	1	Australia	18474	10/10/2014
GCA_003078375.1	ASM307837v1	1	Australia	18474	10/10/2014
GCA_003078415.1	ASM307841v1	1	Australia	18474	10/10/2014
GCA_003078435.1	ASM307843v1	1	Australia	18474	10/10/2014
GCA_003078475.1	ASM307847v1	1	Australia	18474	10/10/2014
GCA_003078835.1	ASM307883v1	N/A[3]	Hisar_India	19141	25/08/2017
GCA_003078815.1	ASM307881v1	16	Hisar_India	19186	17/06/2016
GCA_003077715.1	ASM307771v1	9	Tirupati_India	19063	8/10/2015
GCA_003077755.1	ASM307775v1	10	Tirupati_India	19087	8/10/2015
GCA_003077655.1	ASM307765v1	2	Tirupati_India	19081	8/10/2015
GCA_003077695.1	ASM307769v1	9	Tirupati_India	19039	8/10/2015
GCA_003079075.1	ASM307907v1	4	Pretoria	19188	29/12/2015
GCA_003079115.1	ASM307911v1	3	Pretoria	19186	29/12/2015
GCA_003079155.1	ASM307915v1	10	Pretoria	19188	29/12/2015
GCA_003079235.1	ASM307923v1	9	Pretoria	19177	11/01/2016
GCA_003079255.1	ASM307925v1	11	Pretoria	19185	11/01/2016
GCA_003079695.1	ASM307969v1	8	Pretoria	19195	29/12/2015
GCA_003080895.1	ASM308089v1	9	Pretoria	19179	11/01/2016
GCA_003079415.1	ASM307941v1	N/A[3]	Mittelhaeusern_Switzerland	18462	22/10/2009
GCA_003078895.1	ASM307889v1	N/A[3]	Hyderabad_India	19018	16/07/2012
GCA_003077635.1	ASM307763v1	1	Hyderabad_India	19092	8/10/2015
GCA_003077615.1	ASM307761v1	1	Hyderabad_India	19087	8/10/2015
GCA_003077775.1	ASM307777v1	16	Hyderabad_India	19024	8/10/2015
GCA_003077675.1	ASM307767v1	2	Hyderabad_India	19112	8/10/2015
GCA_003077805.1	ASM307780v1	16	Hyderabad_India	18996	8/10/2015
GCA_003077835.1	ASM307783v1	16	Hyderabad_India	19027	8/10/2015
GCA_003078875.1	ASM307887v1	12	Hyderabad_India	18998	30/12/2013
GCA_003077895.1	ASM307789v1	16	Hyderabad_India	18987	8/10/2015
GCA_003080115.1	ASM308011v1	N/A[3]	Taiwan	19146	23/03/2004
GCA_003173835.1	ASM317383v1	N/A[3]	Greifswald_Germany	18489	13/01/2015
GCA_003080715.1	ASM308071v1	N/A[3]	Guangxi_China	19174	8/02/2018
GCA_003080355.1	ASM308035v1	N/A[3]	Harbin_China	18565	29/02/2016
GCA_003079815.1	ASM307981v1	N/A[3]	Hungary	19186	15/05/2015

GCA_000854445.2	ViralMultiSegProj 14938	N/A[3]	Oxford_UK	19176	21/04/1993
GCF_000854445.3	ViralMultiSegProj 14938	N/A[3]	Oxford_UK	19176	13/07/2004
GCA_003081275.1	ASM308127v1	2	Glasgow_UK	19200	9/09/2011
GCA_003077575.1	ASM307757v1	2	Glasgow_UK	19199	9/09/2011
GCA_003078175.1	ASM307817v1	2	Glasgow_UK	19201	9/09/2011
GCA_003078515.1	ASM307851v1	2	Glasgow_UK	19201	14/11/2013
GCA_003078855.1	ASM307885v1	4	Glasgow_UK	19185	9/09/2011
GCA_003079175.1	ASM307917v1	4	Glasgow_UK	19185	9/09/2011
GCA_003079715.1	ASM307971v1	9	Glasgow_UK	19173	14/11/2013
GCA_003079735.1	ASM307973v1	9	Glasgow_UK	19172	9/09/2011
GCA_003079775.1	ASM307977v1	9	Glasgow_UK	19177	9/09/2011
GCA_003079795.1	ASM307979v1	9	Glasgow_UK	19176	9/09/2011
GCA_003079835.1	ASM307983v1	4	Glasgow_UK	19188	22/07/2014
GCA_003079675.1	ASM307967v1	8	Glasgow_UK	18713	18/07/2014
GCA_003077875.1	ASM307787v1	2	Glasgow_UK	18692	18/07/2014
GCA_003079455.1	ASM307945v1	4	Glasgow_UK	19188	28/08/2014
GCA_003080935.1	ASM308093v1	17	Brazil	19130	29/08/2016
GCA_003080955.1	ASM308095v1	N/A[3]	Pretoria	19168	17/04/2015
GCA_003079975.1	ASM307997v1	N/A[3]	Harbin_China	19187	4/10/2011
GCA_003080215.1	ASM308021v1	1	Harbin_China	19196	23/11/2011
GCA_003081075.1	ASM308107v1	N/A[3]	Cedex_France	19200	17/02/2017
GCA_003081095.1	ASM308109v1	N/A[3]	Cedex_France	19193	17/02/2017
GCA_003081195.1	ASM308119v1	N/A[3]	Cedex_France	19190	17/02/2017
GCA_003081215.1	ASM308121v1	N/A[3]	Cedex_France	19182	17/02/2017
GCA_003081355.1	ASM308135v1	N/A[3]	Cedex_France	19176	6/03/2017
GCA_003081375.1	ASM308137v1	N/A[3]	Cedex_France	19174	17/02/2017
GCA_003080455.1	ASM308045v1	8	Cedex_France	19201	12/02/2016
GCA_003079475.1	ASM307947v1	11	USA	19182	16/06/2015
GCA_003079495.1	ASM307949v1	11	USA	19182	16/06/2015
GCA_003079515.1	ASM307951v1	11	USA	19182	16/06/2015
GCA_003079535.1	ASM307953v1	11	USA	19182	16/06/2015
GCA_003079555.1	ASM307955v1	11	USA	19160	16/06/2015
GCA_003079575.1	ASM307957v1	11	USA	19185	16/06/2015
GCA_003079595.1	ASM307959v1	11	USA	19182	16/06/2015
GCA_003079615.1	ASM307961v1	11	USA	19182	16/06/2015
GCA_003078995.1	ASM307899v1	N/A[3]	Pirbright_UK	19185	2/11/2011
GCA_031522975.1	ASM3152297v1	23	Pirbright_UK	17542	22/01/2013
GCA_003080055.1	ASM308005v1	N/A[3]	Pirbright_UK	19190	4/02/2015
GCA_003078495.1	ASM307849v1	1	Pirbright_UK	19198	1/11/2012
GCA_003078555.1	ASM307855v1	1	Pirbright_UK	19200	1/11/2012
GCA_003078915.1	ASM307891v1	N/A[3]	Hyderabad_India	19133	10/05/2013
GCA_003080415.1	ASM308041v1	11	Belgium	19185	18/07/2012
GCA_003080615.1	ASM308061v1	11	Belgium	19188	18/07/2012
GCA_003080835.1	ASM308083v1	11	Belgium	19185	18/07/2012
GCA_003081055.1	ASM308105v1	11	Belgium	19185	18/07/2012
GCA_003081475.1	ASM308147v1	N/A[3]	Itali	19064	16/12/2016
GCA_003079435.1	ASM307943v1	1	Itali	19192	16/06/2014

GCA_003079015.1	ASM307901v1	1	Itali	19201	16/06/2014
GCA_003079295.1	ASM307929v1	1	Itali	19198	17/07/2014
GCA_003079315.1	ASM307931v1	1	Itali	19198	16/06/2014
GCA_003079335.1	ASM307933v1	1	Itali	19185	16/06/2014
GCA_003079355.1	ASM307935v1	1	Itali	19199	17/06/2014
GCA_003080095.1	ASM308009v1	16	Itali	19200	24/07/2013
GCA_003080235.1	ASM308023v1	3	Itali	18136	23/05/2017
GCA_003080915.1	ASM308091v1	9	Hyderabad,_India	19173	31/01/2012
GCA_031678535.1	ASM3167853v1	8	Israel	18946	19/03/2019
GCA_003077935.1	ASM307793v1	21	Pretoria	19177	17/04/2013
GCA_003079275.1	ASM307927v1	17	Pretoria	19179	17/04/2013
GCA_003080475.1	ASM308047v1	7	Pretoria	19194	17/04/2013
GCA_003080495.1	ASM308049v1	13	Pretoria	19193	17/04/2013
GCA_003080515.1	ASM308051v1	15	Pretoria	19172	17/04/2013
GCA_003080535.1	ASM308053v1	19	Pretoria	19199	17/04/2013
GCA_003080555.1	ASM308055v1	10	Pretoria	19184	17/04/2013
GCA_003080575.1	ASM308057v1	9	Pretoria	19177	17/04/2013
GCA_003080595.1	ASM308059v1	11	Pretoria	19185	17/04/2013
GCA_003080635.1	ASM308063v1	5	Pretoria	19178	17/04/2013
GCA_003080655.1	ASM308065v1	6	Pretoria	19180	17/04/2013
GCA_003080675.1	ASM308067v1	1	Pretoria	19196	17/04/2013
GCA_003080695.1	ASM308069v1	2	Pretoria	19201	17/04/2013
GCA_003080735.1	ASM308073v1	23	Pretoria	19171	17/04/2013
GCA_003080755.1	ASM308075v1	12	Pretoria	19170	17/04/2013
GCA_003080775.1	ASM308077v1	16	Pretoria	19187	17/04/2013
GCA_003080795.1	ASM308079v1	4	Pretoria	19187	17/04/2013
GCA_003080815.1	ASM308081v1	3	Pretoria	19186	17/04/2013
GCA_003080855.1	ASM308085v1	22	Pretoria	19174	17/04/2013
GCA_003080875.1	ASM308087v1	8	Pretoria	19194	17/04/2013
GCA_003080975.1	ASM308097v1	24	Pretoria	19186	17/04/2013
GCA_003080995.1	ASM308099v1	18	Pretoria	19186	17/04/2013
GCA_003081015.1	ASM308101v1	20	Pretoria	19182	17/04/2013
GCA_003081035.1	ASM308103v1	14	Pretoria	19180	17/04/2013
GCA_003080315.1	ASM308031v1	N/A[3]	California_USA	19196	13/06/2012
GCA_003080335.1	ASM308033v1	N/A[3]	Yunnan_China	19172	5/09/2017
GCA_003078715.1	ASM307871v1	N/A[3]	Yunnan_China	19188	8/01/2016
GCA_003080375.1	ASM308037v1	4	Yunnan_China	19177	1/11/2012

N/A[3]= Data not available

Supplementary Table S2. Metadata of the publicly available epizootic hemorrhagic disease virus (EHDV) genome.

**Assembly Accession**	**Assembly Name**	**Serotype**	**Country**	**Assembly Stats Total Sequence Length**	**Assembly Release Date**
GCA_000885335.1	ViralMultiSegProj41081	1	UK	19405	30/09/2009
GCF_000885335.1	ViralMultiSegProj41081	1	UK	19405	9/10/2009
GCA_031522505.1	ASM3152250v1	7	JP	19340	1/10/2022
GCA_031522915.1	ASM3152291v1	1	JP	19300	1/10/2022
GCA_031522965.1	ASM3152296v1	2	CH	19339	12/12/2014
GCA_031322235.1	ASM3132223v1	N/A[4]	USA	19407	10/08/2011
GCA_031522495.1	ASM3152249v1	6	USA	19407	3/01/2019

N/A[4]= Data not available
